# Unlocking new contrast in a scanning helium microscope

**DOI:** 10.1038/ncomms10189

**Published:** 2016-01-04

**Authors:** M. Barr, A. Fahy, J. Martens, A. P. Jardine, D. J. Ward, J. Ellis, W. Allison, P. C. Dastoor

**Affiliations:** 1 Centre for Organic Electronics, University of Newcastle, Callaghan, New South Wales 2308, Australia; 2 Cavendish Laboratory, University of Cambridge, Madingley Road, Cambridge CB3 0HE, UK

## Abstract

Delicate structures (such as biological samples, organic films for polymer electronics and adsorbate layers) suffer degradation under the energetic probes of traditional microscopies. Furthermore, the charged nature of these probes presents difficulties when imaging with electric or magnetic fields, or for insulating materials where the addition of a conductive coating is not desirable. Scanning helium microscopy is able to image such structures completely non-destructively by taking advantage of a neutral helium beam as a chemically, electrically and magnetically inert probe of the sample surface. Here we present scanning helium micrographs demonstrating image contrast arising from a range of mechanisms including, for the first time, chemical contrast observed from a series of metal–semiconductor interfaces. The ability of scanning helium microscopy to distinguish between materials without the risk of damage makes it ideal for investigating a wide range of systems.

## Abstract (web summary)

Scanning helium microscopy uses neutral atoms to image traditionally challenging materials (e.g. delicate, insulating and magnetic samples) non-destructively with absolute surface sensitivity. This work reports the first observation of chemical contrast in helium microscopy via inelastic scattering.

## Graphical abstract

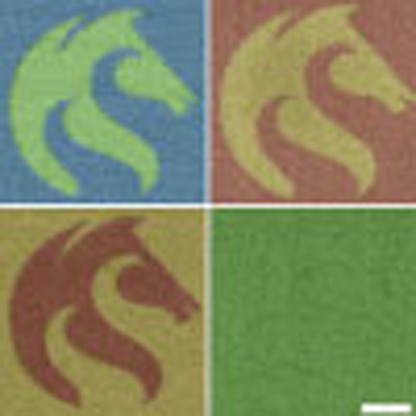

Microscopy is an essential tool for the discovery, application and fabrication of new materials, structures and devices. Moreover, with modern fabrication taking advantage of an ever-broader library of new materials, microscopy techniques need to be applicable to a wide range of organic and inorganic samples^1^,^2^. However, there exists a range of systems that remain challenging to image, such as transparent, fragile, weakly bonded, insulating, very rough and magnetic samples^3^,^4^. Neutral helium atoms are the ideal probe of such systems owing to their low energy, lack of net charge or spin and short de Broglie wavelength. Indeed, the favourable properties of neutral helium atoms as a surface probe have already been exploited for many years in the diffraction-based technique of helium atom scattering (HAS)^4^,^5^. Scanning helium microscopy (SHeM) is a spatially resolved variant of HAS that operates analogously to a scanning electron microscope, with the electron beam replaced with a beam of neutral helium atoms^6^,^7^.

Based on extensive work with HAS, the nature of the probe–sample interaction is well understood at a qualitative level, and research into better quantitative analyses is an active topic in the field of surface scattering^5^,^8^,^9^,^10^,^11^,^12^,^13^. The neutral helium atoms backscatter from the outer electronic corrugation of the sample, thus giving the technique its absolute surface sensitivity and non-destructive qualities. The possible scattering pathways give rise to contrast in the collected image; a critical consideration since useful imaging depends not only on resolution, but on the contrast available. Although the field of atom optics is well established^4^,^14^,^15^,^16^,^17^ and SHeM is already exceeding the resolution limits of traditional optical microscopy^18^, the specific scattering mechanisms by which SHeM image contrast arises is a new area of research. To date, predictions of possible SHeM contrast modes have been purely speculative with no direct comparisons of experimental contrast with theory yet available^4^.

Here we present the first observation of chemical contrast originating from inelastic effects in neutral atom microscopy. SHeM images of different ultrathin patterned metal films on silicon substrates show strong chemical (but weak topological) contrast. Altering the mean energy of the helium beam results in a significant reduction of image contrast, thus providing an unambiguous observation of an inelastic scattering-based process. Finally, we show that current theory is not yet capable of fully explaining the observed contrast.

## Results

### Topological contrast

**Figure 1:**
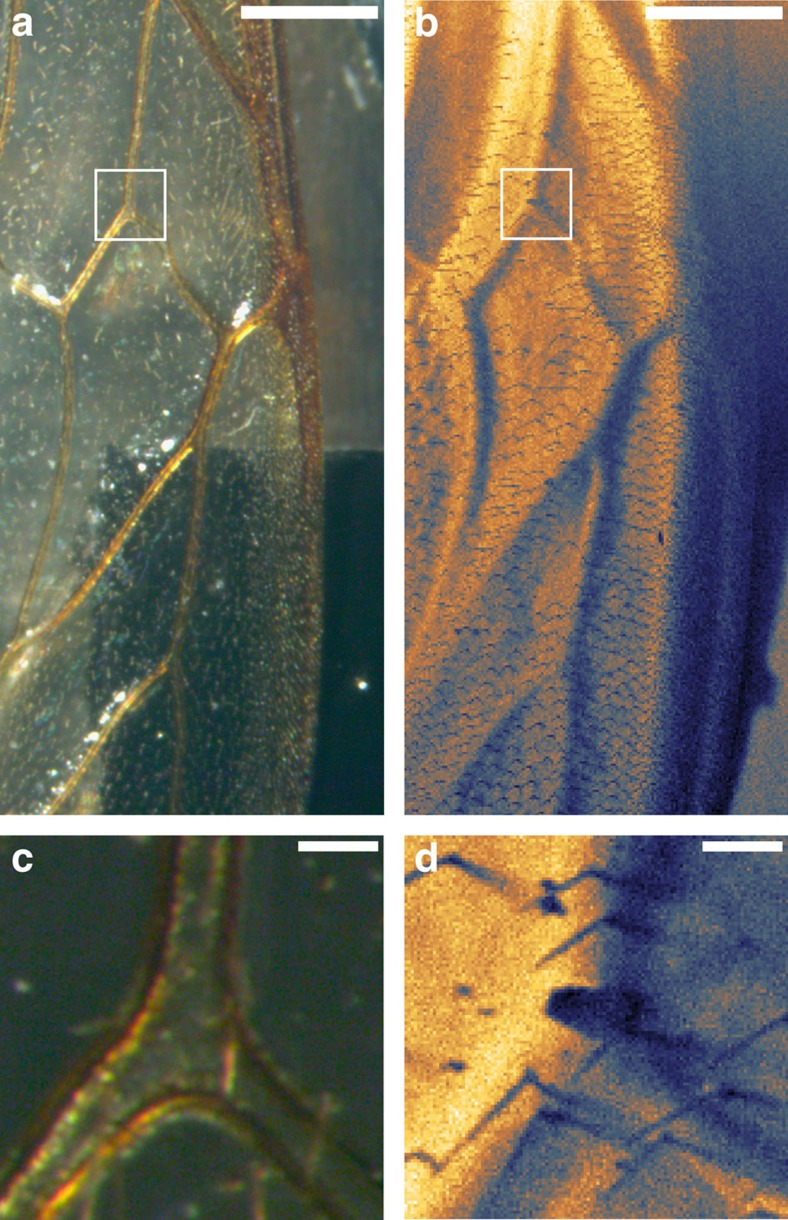
Topological contrast in SHeM. Comparison of reflection optical (Leica M205 C) (**a**,**c**) and SHeM (**b**,**d**) micrographs of a honey bee wing (*Apis mellifera*) as an example of topological contrast. Bottom images taken from the square region are indicated in **a**. Scale bars, 500 and 50 μm, respectively.

Topological contrast is the dominant mechanism in SHeM images of microscopically rough specimens. Deviations from a perfect plane scatter the helium away from the specular channel (‘diffuse scattering'), yielding Michelson contrast *C*:


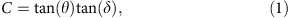


where *θ* is the detector angle and *δ* is the angular mismatch of two scattering planes^4^. As such, changes in the surface morphology will influence the intensity recorded at each pixel of the image. Further topological contrast in the form of shadowing and masking is possible if either the beam or detector is completely occluded due to a surface asperity. Figure 1 shows a comparison of an optical and SHeM micrograph (taken with an instrument detailed previously^7^) of a section of a wing from the honey bee species *Apis mellifera*. The complex folds of the membrane of the wing are the almost indiscernible in the optical image due to the transparency of the membrane material and the range of sample plane heights (in Fig. 1a, the distance from the sample slide to wing top is ∼1.5 mm). However, all of these features are readily apparent in the SHeM image. The absolute surface sensitivity of the helium atoms means that only features on the top side of the wing are observable. For example, masking of the incident helium beam is visible from both the hairs on the wing surface (Fig. 1b) and where the wing rests on the substrate. Thus, SHeM produces intuitive images of biological samples with no sample preparation required and no risk of beam damage to the substrate.

### Chemical contrast

While topological contrast is readily observed, the properties of the helium probe particle have been predicted to yield weaker, more exotic mechanisms^4^. For example, the composition and local atomic character of a sample surface should also give rise to variations in the helium reflectivity through the structure factor of the scattering centres. The mean energy and momentum of a neutral helium beam with a de Broglie wavelength of the order Ångstroms is well-matched to those of phonon-induced surface charge–density oscillations, making it capable of interacting with dynamic surface processes. Indeed, an atomic beam of helium is ideal to probe such processes since its small size and mass (as compared to heavier noble species) minimizes the lattice displacement and hence helium atoms will excite or de-excite the largest number of vibrational modes^8^,^19^. Traditionally, the Debye–Waller factor (DWF) is used to describe the variation in specular (‘in-plane') reflectivity (*I/I*_*0*_) via interactions with phonons. One definition of the factor has the form:


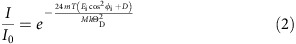


and describes an atomic beam of energy *E*_i_, mass *m*, incident on a surface of atomic mass *M* at angle *φ*_i_, temperature *T* and Debye temperature *Θ*_D_. *D* is the potential well depth of the interaction of the helium atom with the surface and *k* is the Boltzmann constant^5^. The prevalence of the vibrations can thus be seen to be highly dependent on sample composition. For example, a material with a rigid lattice or high molecular mass will divert less of the helium signal away from the specular channel (‘out-of-plane' scattering). In addition, the presence of adsorbates on the sample surface does not necessarily prevent such interactions since the surface charge density is impacted by the motion of atoms buried deep below the surface and the incident helium atoms can probe these subsurface resonances^9^,^10^. In principle, therefore, the helium–electron–phonon coupling can provide chemical contrast with which to characterize a surface even in the presence of multiple adsorbate layers.

**Figure 2:**
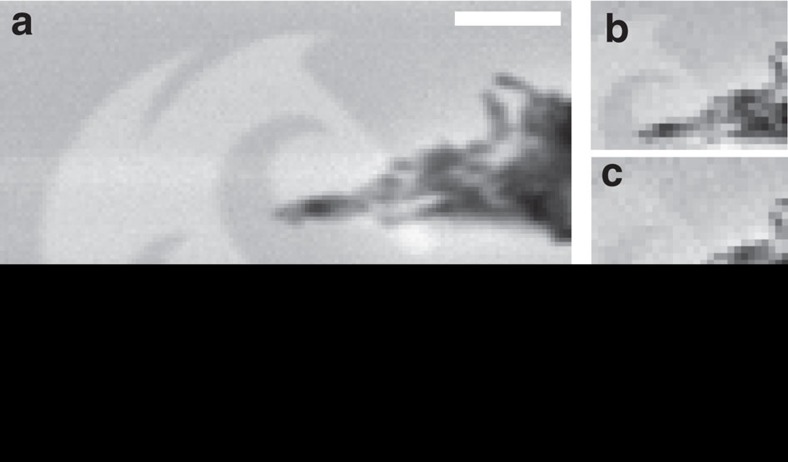
Comparison of contrast mechanisms accessible by SHeM. Micrographs of a gold University of Newcastle logo partially obscured by a piece of dust. (**a**) Full image taken with the sample at specular, while **b** shows a section of the sample at the same position. (**c**,**d**) Small region of the sample at 500 and 1,000 μm further back from the pinhole respectively. Scale bar, 50 μm.

HAS studies have demonstrated that the helium signal that is inelastically scattered is small in comparison with the elastic contribution—typically 2–3 orders of magnitude lower^8^,^19^. To achieve an unambiguous observation of any chemical contrast effects, the materials and sample geometry were carefully selected to minimize any competing contrast mechanisms. Using electron beam lithography, a 15 nm thick patterned layer of gold was laid down on a silicon (100) substrate with a native oxide layer; a 3 nm titanium layer was added first to facilitate the wetting of the silicon surface. Figure 2a shows a SHeM micrograph of the metal on silicon sample, with a topological feature provided by a dust particle, which had come to rest on part of the gold layer. As can be clearly seen in Fig. 2, the nanometre-thin metal layer is visible against the silicon substrate, but the contrast is much weaker than the topological contrast; consistent with the expected result for an inelastic process. Figure 2b–d show the image progression as the gold on silicon sample was moved back from the specular position in 500 micron increments. Although the detector acceptance angle in the current apparatus is large, the distance moved is sufficient to place the sample specular position outside the detector's acceptance cone. While the contrast between the gold and silicon diminishes with increasing sample distance from the specular position, there is no trend in the contrast between the dust particle and underlying silicon as a function of sample position. This observation indicates that while the contrast in the dust particle is purely topological in nature, the change in contrast for the gold logo cannot be due to a simple step height or mean plane topological feature. It is possible that a consistent difference in surface roughness between the gold and silicon (on a scale much smaller than the instrument spot size) could explain the observed inter-material contrast. To eliminate this possibility, atomic force microscope (AFM) studies were conducted using a Cypher scanning probe microscope with sub-nanometre resolution in both lateral and axial directions to determine the degree of surface irregularity in both materials. It was found that the gold had a root mean square (r.m.s.) roughness of 2.8 nm, while the silicon yielded a value of 1.2 nm. The level of observed roughness is orders of magnitude smaller than the beam spot size and thus it seems unlikely that this difference in roughness is the cause of the observed contrast. Furthermore, a coarser surface would (in general) be expected to cause a higher degree of diffuse scattering away from specular, and thus would appear darker in a SHeM micrograph; the inverse to the observed results in Fig. 2.

**Figure 3:**
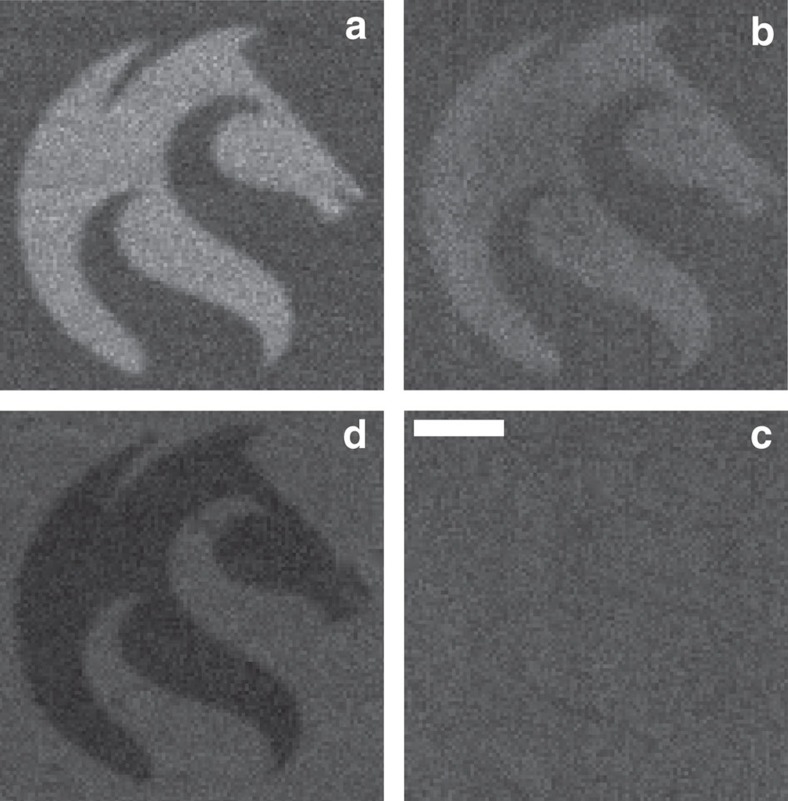
Metal–semiconductor interfaces as imaged using neutral helium. SHeM micrographs show the University of Newcastle logo in different metals on a silicon substrate. Clockwise from top left: (**a**) gold (**b**) nickel (**c**) platinum and (**d**) chromium. Scale bar, 50 μm.

To determine whether contrast could be observed with other metal/semiconductor combinations, 40-nm-thick patterned layers of gold, chromium, nickel and platinum were laid down on the same silicon substrate with a 3 nm titanium wetting layer, in the same manner as the prior 15 nm gold sample. Figure 3 shows a composite image of the different metallic layers under SHeM. It should be noted that in each of the component images in Fig. 3, the incident helium intensities were normalized using the mean silicon signal as the reference. The SHeM images clearly show that there is distinct contrast between each metal and the silicon substrate as well as between the metal species. The mean r.m.s. roughness of the gold, nickel, platinum, chromium and silicon measured by AFM was 2.1, 0.9, 1.6, 2.6 and 1.4 nm, respectively. Given that the observed contrast does not follow the roughness trend in the AFM data, and that the same wetting layer was used for each sample, it seems unlikely that changes in diffuse scattering arising from the nanometre scale surface irregularities can explain the contrast differences in Fig. 3.

**Figure 4:**
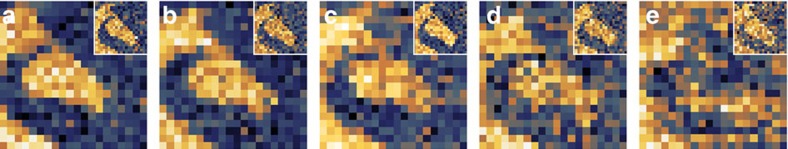
Effect of varying helium mean beam energy on sample contrast. (**a**–**e**) 83, 72, 66, 42, and 21 meV SHeM micrographs of the 15-nm-thick gold on silicon sample. All images were taken with the sample at the specular position, with a 200 bar beam and the sample at room temperature (294 K). Insets show simulated images with Poisson noise added, according to the observed count rates. Note that the contrast is maintained in the simulations, but lost in the data, which confirms the presence of an additional contrast mechanism.

To investigate the source of the contrast further, the energy of the incident helium was varied by either warming or cooling the stagnation volume of our supersonic free-jet expansion. The 15 nm gold on silicon sample was scanned with a range of beam energies (as shown by the series of images in Fig. 4) with Michelson contrast observed to diminish with the reduction in energy. As a control study, a copper transmission electron microscope grid on a silicon wafer substrate was imaged across the same range of mean beam energies, with no trend in Michelson contrast observed. The SHeM is currently limited by the detection count rate through Poisson statistics, and thus changes in beam intensity (and hence signal:noise ratio) as a function of beam energy also influence image quality. However, simulated SHeM micrographs based on the contrast observed at room temperature and matched to the experimentally observed signal-to-noise ratio at each beam energy could not explain the reduction in image quality (Fig. 4 inset). Moreover, the simulation was only able to replicate the data set through a direct reduction in material contrast as a function of temperature.

The angular distribution of scattered helium, which controls contrast in the SHeM, is related to the local atomic scale character of the surface convolved with the beam spot and detector aperture^7^. The local vibrational modes influence the degree of inelastic scattering, while the average structural form factor of the scatterers controls diffuse elastic scattering. While inelastic scattering is strongly dependent on beam energy (equation 2), diffuse elastic scattering varies only weakly through the much smaller associated change in momentum of the beam. Hence, given the large reduction in contrast as a function of decreasing beam energy observed in Fig. 4, we can conclude that the dominant contrast displayed by the metal–silicon interface samples is a consequence of inelastic processes; that is, helium–electron–phonon interactions.

## Discussion

Using the DWF as a starting point, one can attempt to predict the expected contrast due to lattice vibrations. Using the model presented by MacLaren *et al.*^4^, it was found that while the contrast between the nickel and platinum in Fig. 3 qualitatively matched the model, the contrast between the gold and chromium was found to be inverted from that predicted. This result is perhaps not unexpected; early work with the scattering of helium from surfaces (which attempted to verify the DWF for gas–surface interactions) produced consistent agreement with the DWF for some systems, yet for others it was wholly inadequate^11^,^12^. Attempts have been made in recent times to redefine the DWF in terms of quantum gas–surface interactions^13^, but it is clear that the complex vibrational behaviour of materials remains an active area of research within the field of surface scattering. It is also important to note that these observations do not rely on the specific form of the metal surface layer. The metals utilized in this study are either noble metals, or form thin, passivating oxide layers and as such were chosen to reduce the complexity of any subsequent comparisons to theory. Nevertheless, oxide or other physisorbed layers of contamination will certainly be present in our *ex situ* prepared samples. However, it is clear that chemical contrast from the underlying material is still evident in the micrographs in Figs 2, 3, 4. Work in this laboratory is underway to allow the addition of a low-damage sample-cleaning method to the SHeM instrument to create the ultraclean, well-ordered systems needed to examine the origins of both surface and subsurface contrast in more detail. Modelling the interaction of helium with even these ideal systems remains an open problem. Indeed, the development of SHeM provides both a new opportunity, and an impetus, for further research into the complex vibrational behaviour of materials.

Thus far, we have shown that by utilizing existing detector technology and simple atom optics, the current SHeM instrument demonstrates both topological and chemical contrast. However, looking ahead there is a further contrast mechanism that is, in principle, available to the SHeM instrument. Under normal scattering conditions, thermal helium atoms have a de Broglie wavelength comparable to the typical crystallographic dimensions. As a result, the backscattered helium atoms produce diffraction patterns characteristic of the surface corrugation potential to yield information about the surface structure. To resolve these diffraction patterns, typical HAS apparatus have an angular resolution of less than half a degree^5^. With a working distance of 2.8 mm, the current SHeM instrument has a beam spot size of 5.4 μm and detector acceptance angle of 14.5 degrees. The measured intensity involves a convolution of the atomic form factor spatially with the beam spot, and angularly with the acceptance angle, therefore, precluding the observation of individual diffraction peaks. Consequently, diffractive effects do not play a role in the generated contrast. Furthermore, it is well-known from prior HAS studies^5^ that even small amounts of disordered adsorbates lead to a reduction in the intensity of the helium diffraction peaks, and so sample cleaning would be a necessary requirement. To adapt the SHeM to achieve HAS-like angular resolution, a 3–4 order of magnitude increase in detector sensitivity is required (based on the reduction in detector aperture size), which can be realized by utilizing a solenoidal ion source^20^,^21^. Enhanced angular resolution would give the SHeM access to an imaging mode analogous to the dark field imaging of electron microscopy. The ability to resolve the local order of surfaces, even when composed of the same material, would provide critical insight into a number of current surface science investigations. An example includes the study of organic photovoltaics, where it has been shown that the crystallinity of the polymers at interfaces within the device directly affects performance^22^.

In summary, the experiments presented in this paper demonstrate that contrast in SHeM arises not only due to surface topology, but also the local chemical environment through inelastic interactions. Furthermore, we have clearly shown that the observed contrast does not quantitatively match present theoretical models due to the complexity of helium–electron–phonon coupling. The observation of chemical contrast in SHeM offers the prospect of new theoretical studies in this field. Most importantly, based on the technique's surface sensitivity, non-destructive inert nature, potential for nanometre resolution and range of novel contrast mechanisms, SHeM is already able to be applied to a large variety of systems as a complimentary technique to the existing surface analysis tool set.

## Methods

### Operation of the Mk II SHeM

**Figure 5:**
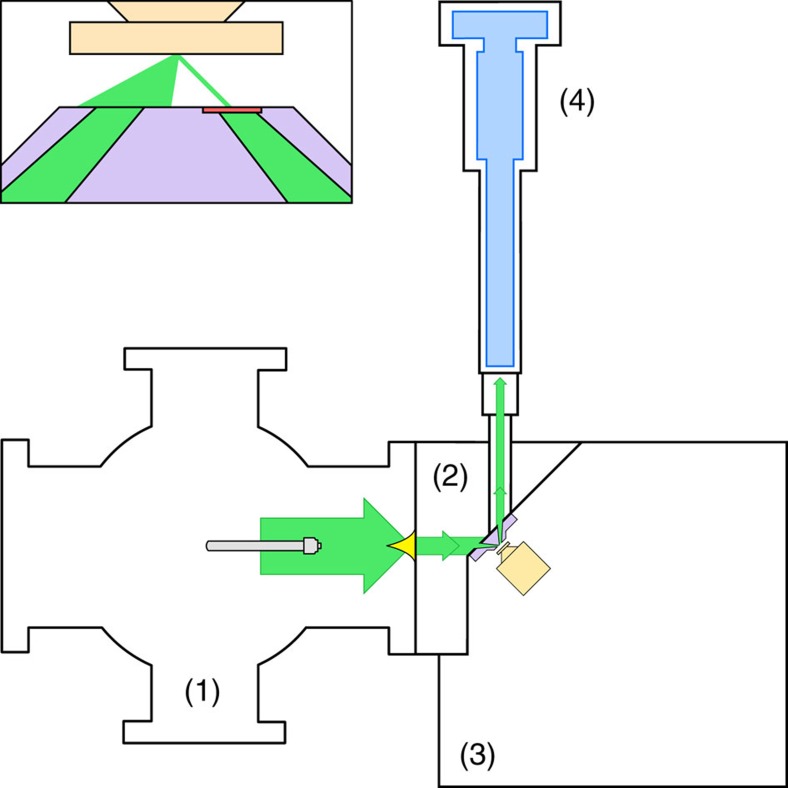
Schematic diagram of the Mk II scanning helium microscope. Inset shows the pinhole plate with focused ion beam-milled pinhole used to aperture the beam.

Figure 5 shows a schematic diagram of the Mk II SHeM used in this study^7^. In the source chamber (1), a helium free-jet beam expansion (maximum pressure of 240 bar, temperature adjustable from 100 to 400 K) is created with the aid of a 10 micron nozzle, before the centreline of the expansion is selected out by a 100 micron skimmer (Beam Dynamics, Inc., model 2). The resultant helium beam enters the differential stage (2), where it is progressively apertured by the pinhole plate. In the tip of the pinhole plate a silicon nitride disc (Ted Pella part no. 21525) with a focused ion beam-milled pinhole forms the final optical element, leaving a small spot to strike the sample surface in the sample chamber (3), as shown in the inset. The helium backscattered from the sample enters the detector chamber (4) through a 1-mm diameter aperture, where it stagnates to form a stable pressure, subsequently measured by a Hiden HAL/3F PIC mass spectrometer. By rastering the sample back and forth, an image of the surface may be constructed. With the aim of the work presented here being to investigate the contrast available to the technique, a 5-μm pinhole was utilized in all images shown to keep the count rates high.

## Additional information

**How to cite this article:** Barr, M. *et al.* Unlocking new contrast in a scanning helium microscope. *Nat. Commun.* 7:10189 doi: 10.1038/ncomms10189 (2016).
